# Comparison of the Kinetics of Maturation of Phagosomes Containing Apoptotic Cells and IgG-Opsonized Particles

**DOI:** 10.1371/journal.pone.0048391

**Published:** 2012-10-31

**Authors:** Michelle S. Viegas, Luís M. B. B. Estronca, Otília V. Vieira

**Affiliations:** CNC - Center for Neuroscience and Cell Biology, University of Coimbra, Largo Marquês de Pombal, Coimbra, Portugal; Duke University Medical Center, United States of America

## Abstract

Defective clearance of apoptotic cells has emerged as an important contributing factor to the pathogenesis of many diseases. Although many efforts have been made to understand the machinery involved in the recognition between phagocytes and potential targets, little is known about the intracellular transport of phagosomes containing apoptotic cells within mammalian cells. We have, therefore, performed a detailed study on the maturation of phagosomes containing apoptotic cells in a non-professional phagocytic cell line. This process was compared with the maturation of IgG-opsonized particles, which are internalized via the Fcγ-receptor (Fcγ-R), one of the best characterized phagocytic receptor, in the same cell line stably expressing the Fcγ-RIIA. By comparing markers from different stages of phagosome maturation, we have found that phagosomes carrying apoptotic particles reach the lysosomes with a delay compared to those containing IgG-opsonized particles. Enrichment of the apoptotic particles in phosphatidylserine (PS) neither changed the kinetics of their engulfment nor the maturation process of the phagosome.

## Introduction

Phagocytosis is a complex cellular event by which large particles are actively recognized, engulfed and degraded. Although, the predominant focus has been on the role in host defense, this process also plays a critical role in removal of apoptotic cells that is essential for tissue remodeling and homeostasis [1]–[4]. This is particularly important in diseases such as atherosclerosis and neurodegenerative diseases. In both cases, professional phagocytes are not the only players involved. The removal of apoptotic cell debris in atherosclerosis, for example, is known to specifically involve smooth muscle cells (SMC) that are not professional phagocytes to a very significant degree. In fact, in atherosclerosis, SMCs represent the major phagocytic population in the vessel wall besides macrophages [5]–[7].

The past decade has seen an impressive expansion on our knowledge regarding the fundamentals of apoptotic cell clearance. Based on work from many laboratories, several broadly defined steps have been identified in the recognition and removal of apoptotic cells by phagocytes. Each step appears to be tightly regulated by signaling events to ensure swift and efficient clearance. At the early stage of apoptosis, the dying cells release “find-me” signals that are sensed by motile phagocytes and attract them to the proximity of the dying cell. Several of these soluble chemoattractant find-me signals released during apoptosis have been recently identified [8]–[11]. The physical contact between the apoptotic cell and the phagocyte is mediated via ligands on the apoptotic cell (referred to as “eat-me” signals) and engulfment receptors on phagocytes that can recognize these eat-me markers. Among the array of identified eat-me molecules [12], the exposure of PS on the outer leaflet of the apoptotic cell plasma membrane appears to be a key marker [13], [14]. This lipid, normally concentrated on the inner leaflet of the plasma membrane, loses its asymmetric distribution during apoptosis and is translocated to the outer leaflet of the plasma membrane [13], [15]. Phagocyte recognition of PS is mediated directly via one or more PS receptors, including BAI-1, Tim-4 and Stabilin-2 [16]–[21], or by bridging molecules, that bind PS on the apoptotic cell and a receptor on the phagocyte [22]–[24]. For some of these receptors direct or indirect ligation to PS results in Rac-dependent cytoskeletal reorganization, which ultimately leads to engulfment of the apoptotic cell [12]. Once inside the phagocyte, the ingested apoptotic cargo is processed via a phagolysosomal pathway that shares features with the endocytic machinery but has some unique features of its own [25]–[29]. Specifically, the phagosomal membrane initially acquires markers of early endosomes, these are subsequently lost from the phagosome and are replaced by markers of late endosomes. Ultimately, lysosomal contents (e.g. cathepsins) and membrane constituents (e.g. LAMP-1, also present in late endosomes) are found in a terminal hybrid organelle, the phagolysosome. This sequence is accompanied by a progressive acidification of the phagosomal lumen, that correlates with the acquisition of vacuolar H^+^-ATPases and recycling of phagosomal components [30]–[33].

Most of the literature in mammalian systems has addressed uptake and maturation of apoptotic cells by macrophages (or immature dendritic cells), but there are other cell types such as fibroblasts, epithelial, endothelial and smooth muscle cells, among others that are responsible for mediating the clearance [2]. It has been increasingly suggested that there are important distinctions in the molecular mechanisms underlying phagocytosis by different receptors, originating different phagosomes. The focus has been on differences in cytoskeletal elements that mediate ingestion, in vacuole maturation and in the inflammatory responses generated [1]. However, the understanding of the molecular processes that underlie maturation of apoptotic cells-containing phagosomes is rudimentary, thus any interruption in this finely tuned system can eventually progress to a post-apoptotic secondary necrotic state, inflammation and to the onset of several diseases, such as: atherosclerosis, autoimmunity and neurological diseases [34]–[36]. In this context, we decided to address, in detail, the interaction of nascent phagosomes containing apoptotic cells with components of the endocytic pathway, recycling of phagosomal components and lumenal acidification and compare this process with the maturation of phagosomes containing IgG-opsonized particles that are engulfed via the Fc-receptors. These studies were performed in a non-professional phagocytic Smooth Muscle Cell line (SMC). To exclude interference of the cell type in our comparative study, we also generated SMC stably expressing Fcγ-RIIA. We specifically chose these cells because one of our major research interests is to understand why the uptake of apoptotic cells in atherosclerosis is impaired. As phagocytic particles, we used aged human red blood cells (agRBC) as a model of apoptotic cells and opsonized sheep red blood cells (shRBC) or latex beads (which are internalized via the widely studied Fcγ-R).

We report here that maturation of phagosomes containing apoptotic cells mature slower than phagosomes containing IgG-opsonized RBCs in SMCs. Furthermore, we addressed whether agRBC loaded with PS, the best known “eat-me” signal on the cell surface of apoptotic cell, affected phagocytosis and phagosomal maturation. Our results show that PS loading of our apoptotic cell model affects neither phagocytosis nor phagolysosome formation.

## Materials and Methods

### Antibodies and Dyes

Goat anti-EEA-1 and mouse anti-LBPA antibodies were from Echelon Bioscience Inc. and Santa Cruz Biotechnology, respectively. Rabbit anti-sheep red blood cells was from MP Biomedicals Inc. Secondary fluorescent antibodies were from Molecular Probes or from Jackson Immunoresearch. Rhodamine-Phalloidin, LysoTracker Red DND-99, Dextran Tetramethylrhodamine conjugate (10,000 MW, lysine fixable, fluoro-ruby), CellTrace CFSE; and CellTracker Orange CMTMR were from Molecular Probes. DAPI was from Fluka.

**Figure 1:**
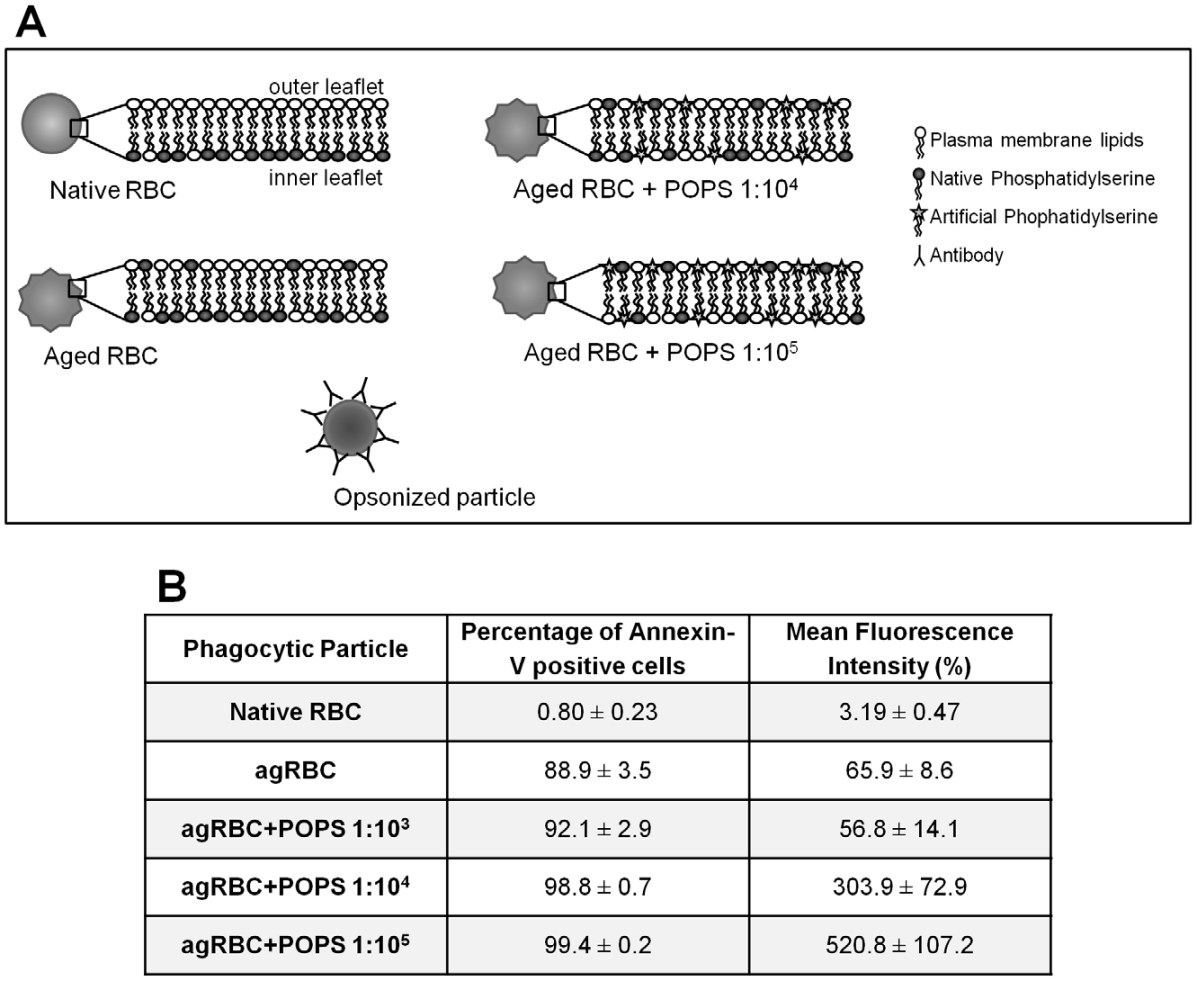
Enrichment of agRBC in phosphatidylserine. A ) Schematic representation of the different phagocytic models used in the experimental work. **B**) Flow cytometry analysis of agRBC labeled with Annexin V-FITC before and after overnight incubation with different cell:PS liposome ratios to enrich the outer leaflet of the agRBC plasma membrane with PS, as described in Experimental Procedures. The table shows the percentage of Annexin V-positive RBCs and Mean Fluorescence Intensity ± SEM of at least three independent experiments. A total of 20.000 cells were analyzed in each condition.

### RBC Isolation and Aging

Red blood cells from human blood were isolated in a Ficoll-Paque gradient, washed with PBS and then resuspended at 20% hematocrit in PBS with 0.1% glucose. Human blood was obtained from healthy volunteers at Center for Neuroscience and Cell Biology. Written informed consent was obtained from all volunteers, who signed informed consent forms for this purpose, approved by the Ethical Review Board of the Faculty of Medicine of the University of Coimbra. Sheep red blood were isolated and used as described before [37]. These cells were kept at 4°C and used as native RBCs. AgRBC were prepared by incubating native RBCs in PBS (20% hematocrit) at 37°C for 4 days. Loss of phospholipid asymmetry of agRBC was assessed by flow cytometry (FACScalibur, Becton Dickinson) using Annexin V-FITC (BD Bioscience). Briefly, for these experiments, RBCs were washed twice with cold PBS, and 10^6^ cells were re-suspended in HEPES buffer pH 7.4 containing 2.5 mM calcium for 15 min at room temperature (RT) in the dark with 5 µL of Annexin V-FITC solution (BD Biosciences, Pharmingen). Following washing, cells were gated for biparametric histograms FL1 versus FL2.

**Figure 2:**
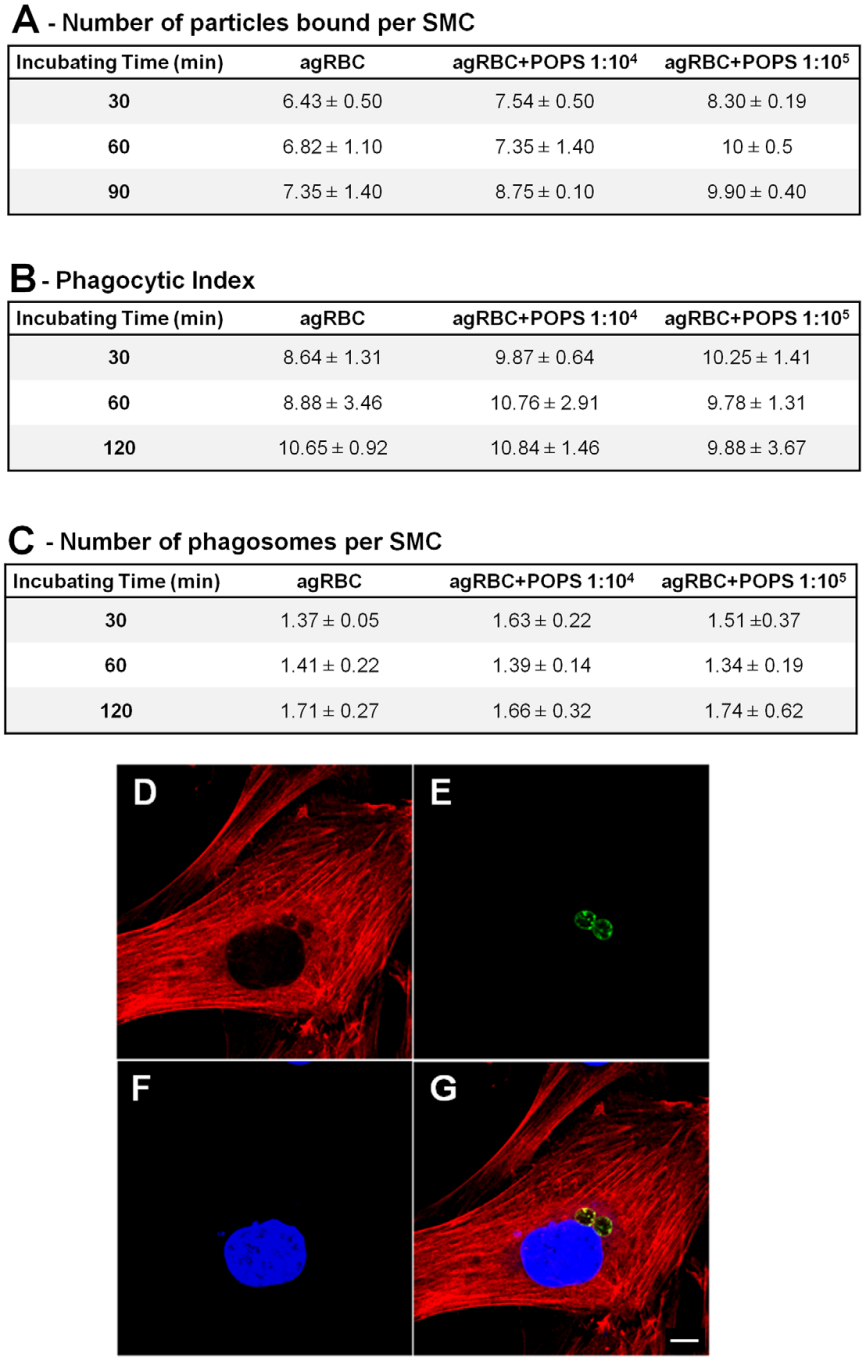
Effect of PS enrichment in binding and phagocytosis of agRBC by SMC. After aging and their incubation with different ratios of RBC:PS the cells were labeled and added to the SMC. **A**) Effect of PS enrichment in binding of the agRBC to SMC as a function of time. SMC were allowed to bind RBC on ice, and then warmed up for 35 s as described in Experimental Procedures. The cells were then fixed and analyzed by light microscopy. The values show the number of RBCs bound per 100 phagocytic cells. **B**) Effect of PS enrichment in phagocytosis of agRBC. The values show the percentage of SMC that have internalized phagocytic particles (Phagocytic Index). **C**) Effect of PS enrichment in the number of RBC ingested per phagocytic cell. Results are expressed as mean ± SD of at least three independent experiments. At each time point 100 phagosomes were analyzed. **D-G**) Representative images of agRBC phagocytosis by a SMC at 0 min chase time. **D**) Actin staining with Rhodamine-Phalloidin (in red). **E**) Engulfed agRBCs stained with CFSE (in green). **F**) Nucleus staining with DAPI (in blue). **G**) Corresponding merged image. Bar, 10 µm.

### Preparation of PS Liposomes and its Incorporation into agRBC

Aqueous suspensions of 1-palmitoyl-2-oleoyl-sn-glycero-3-phospho-L-serine (PS), obtained from Avanti Polar Lipids Inc., were prepared by adding to the lipid powder the hydration solution (0.11 M NaCl, pH 7.4) in a water bath at 65°C. The samples were submitted to several cycles of vortex/incubation at 65°C for at least 1 h. The resulting multilamellar vesicle suspension was extruded through two stacked polycarbonate filters (Nucleopore) with a pore diameter of 0.1 µm using a minimum of 10 passages. During the extrusion the water-jacketed extruder (Lipex Biomembranes, Vancouver, British Columbia, Canada) was maintained at 65°C. After extrusion the lipids were sonicated for 1 h and quantification of phospholipid concentration was done by a modified version of Bartlett’s phosphate assay [38]. Then, agRBC were incubated overnight with POPS liposomes at different RBC:PS (cell:lipid) ratios (1∶10^3^, 1∶10^4^ and 1∶10^5^). The incorporation of PS into the outer leaflet of the plasma membrane of agRBCs was also assessed by flow cytometry with Annexin V-FITC as described before.

**Figure 3:**
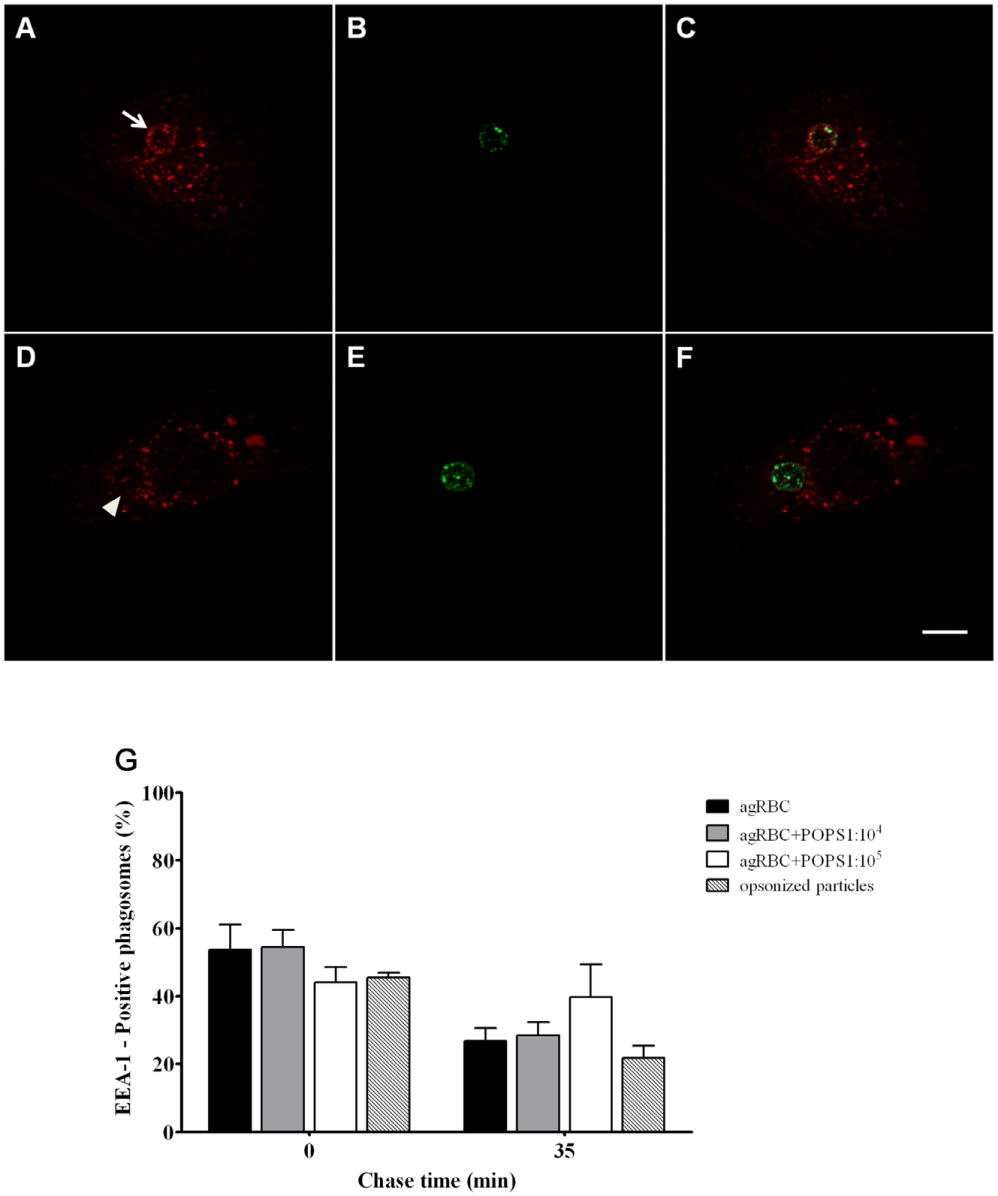
Interaction of phagosomes containing different phagocytic particles with early endosomes. The interaction of early endosomes with phagosomes containing different particles was assessed by the acquisition of EEA-1. After the pulse-chase experiments, the cells were fixed, stained with EEA-1 antibodies and analyzed under a confocal microscope. **A**) EEA-1 staining of a cell containing an EEA1-positive phagosome. **B**) Internalized agRBC stained with CFSE. **C**) Corresponding merged image. **D**) EEA-1 staining of a cell containing an EEA1-negative phagosome. **E**) Internalized agRBC stained with CFSE. **F**) Corresponding merged image. Arrow indicates a positive phagosome at 0 min chase time and arrowhead indicates a negative phagosome after 35 min chase time. Bars, 10 µm. **G**) Quantification of the EEA1-positive phagosomes. Wild type and engineered SMCs were exposed to different phagocytic particles for 30 min and then chased for the time indicated in the graph abscissa. The values are means ± SEM of, at least, three independent experiments. At each time point 100 phagosomes were analyzed.

### Cell Culture and Generation of SMC Stably Expressing the Fcγ-RIIA

Rabbit femoral smooth muscle cells (SMC) were from ATCC (Camden, NJ, USA) and were maintained in RPMI-1640 medium supplemented with 10% fetal calf serum, 100 U/mL of penicillin and streptomycin. Cells were grown in a humidified incubator at 37°C under 5% CO_2_ and used for assays during the exponential growth phase.

**Figure 4:**
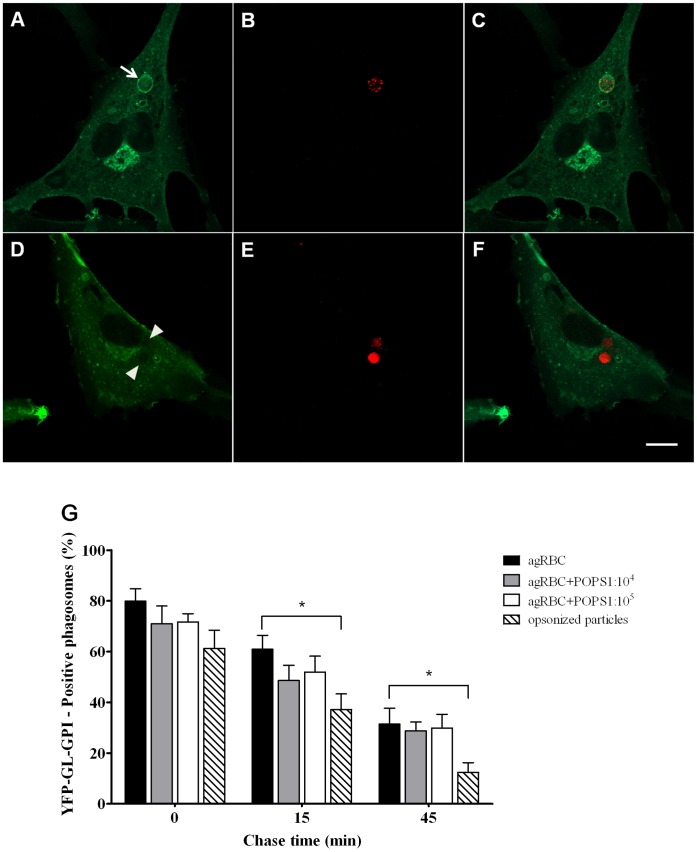
Recycling of YFP-GL-GPI from phagosomal membranes containing different phagocytic particles. The recycling of plasma membrane components from phagosomal membranes was assessed by the loss of the plasma membrane marker YFP-GL-GPI. Wild-type and engineered SMCs were infected with adenoviruses expressing YFP-GL-GPI. **A**) Cell expressing YFP-GL-GPI and containing a YFP-GL-GPI-positive phagosome at 0 min chase time. **B**) Internalized agRBC stained with CMTMR. **C**) Corresponding merged image. **D**) SMC expressing the FcγR-IIA and YFP-GL-GPI showing a negative phagosome for YFP-GL-GPI at 45 min chase time. **E**) Internalized IgG-shRBC stained with CMTMR. **F**) Corresponding merged image. Arrow indicates an YFP-GL-GPI-positive phagosome and arrowheads indicates two YFP-GL-GPI-negative phagosomes. Bars, 10 µm. **G**) Quantification of the YFP-GL-GPI-positive phagosomes. Wild-type and engineered SMCs expressing YFP-GL-GPI were exposed to different phagocytic particles for 30 min and then chased for the times indicated in the graph abscissa. The results are means ± SEM of, at least, three independent experiments. Samples were analyzed by fluorescence confocal microscopy. At each time point 100 phagosomes were analyzed. *, p < 0.05 comparing differences between loss of YFP-GL-GPI by phagosomes with agRBC and with IgG-opsonized RBCs.

In order to generate SMC stably expressing Fcγ-RIIA, the Human Fcγ-RIIA tagged with myc was subcloned into the retroviral vector pBABE-puro. The retrovirus production, cells infection and selection were done as described before [37], [39]. In both cases, cells were used between 1 and 8 passages.

**Figure 5:**
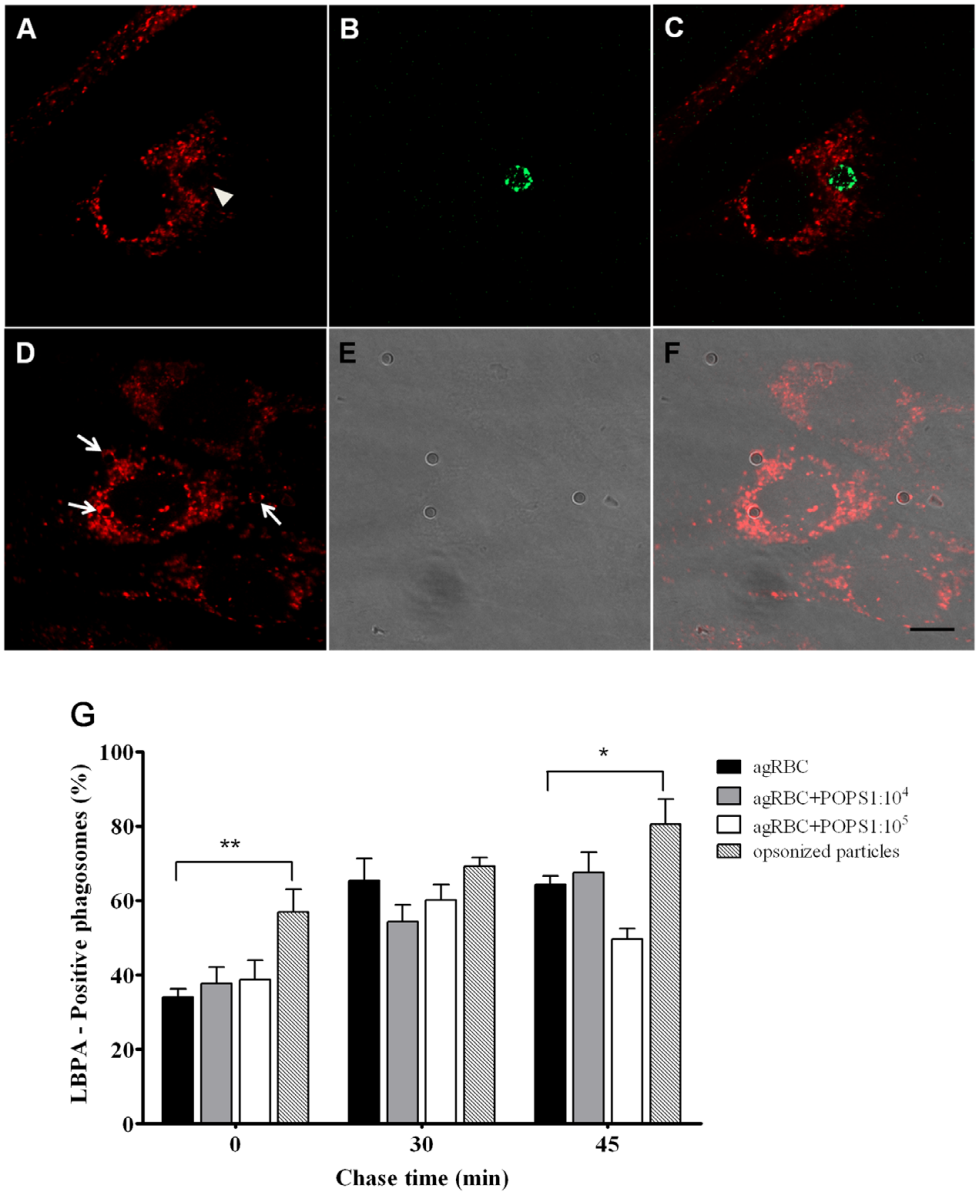
Interaction of phagosomes containing different phagocytic particles with MVB/Late endosomes. LBPA acquisition was used to assess the interaction of phagosomes containing different particles with MVB/late endosomes. **A**) LBPA staining of a cell containing a LBPA-negative phagosome. **B**) Internalized agRBC stained with CFSE. **C**) Corresponding merged image. **D**) LBPA staining of a cell stably expressing the FcRγ-IIA and containing three LBPA-positive phagosomes. **E**) Corresponding differential interference contrast (DIC) image. **F**) Corresponding merged image. Arrows indicate three LBPA-positive phagosomes and arrowhead indicate a negative phagosome after 30 min pulse (0 min chase time). Bars, 10 µm. **G**) Quantification of the LBPA-positive phagosomes. Wild type and engineered SMCs were exposed to different phagocytic particles for 30 min and then chased for the times indicated in the graph abscissa. After fixation the cells were stained with LBPA antibodies and analyzed under a confocal microscope. Data shows the percentage of LBPA-positive phagosomes and are means ± SEM of, at least, three independent experiments. At each time point 100 phagosomes were analyzed. *, p < 0.05 and **, p < 0.01 comparing differences between LBPA-acquisition by phagosomes with agRBC and with IgG-opsonized latex beads.

### Binding, Phagocytosis and Phagosomal Maturation Assays

Before incubation with PS liposomes or IgG-opsonization, RBC were labeled with carboxyfluorescein-diacetate-succinimidyl ester (CFSE) or Orange-CMTMR, at final concentration of 0.5 µM, for 15 min at 37°C. The shRBC and the latex beads were opsonized as described before [38].

**Figure 6:**
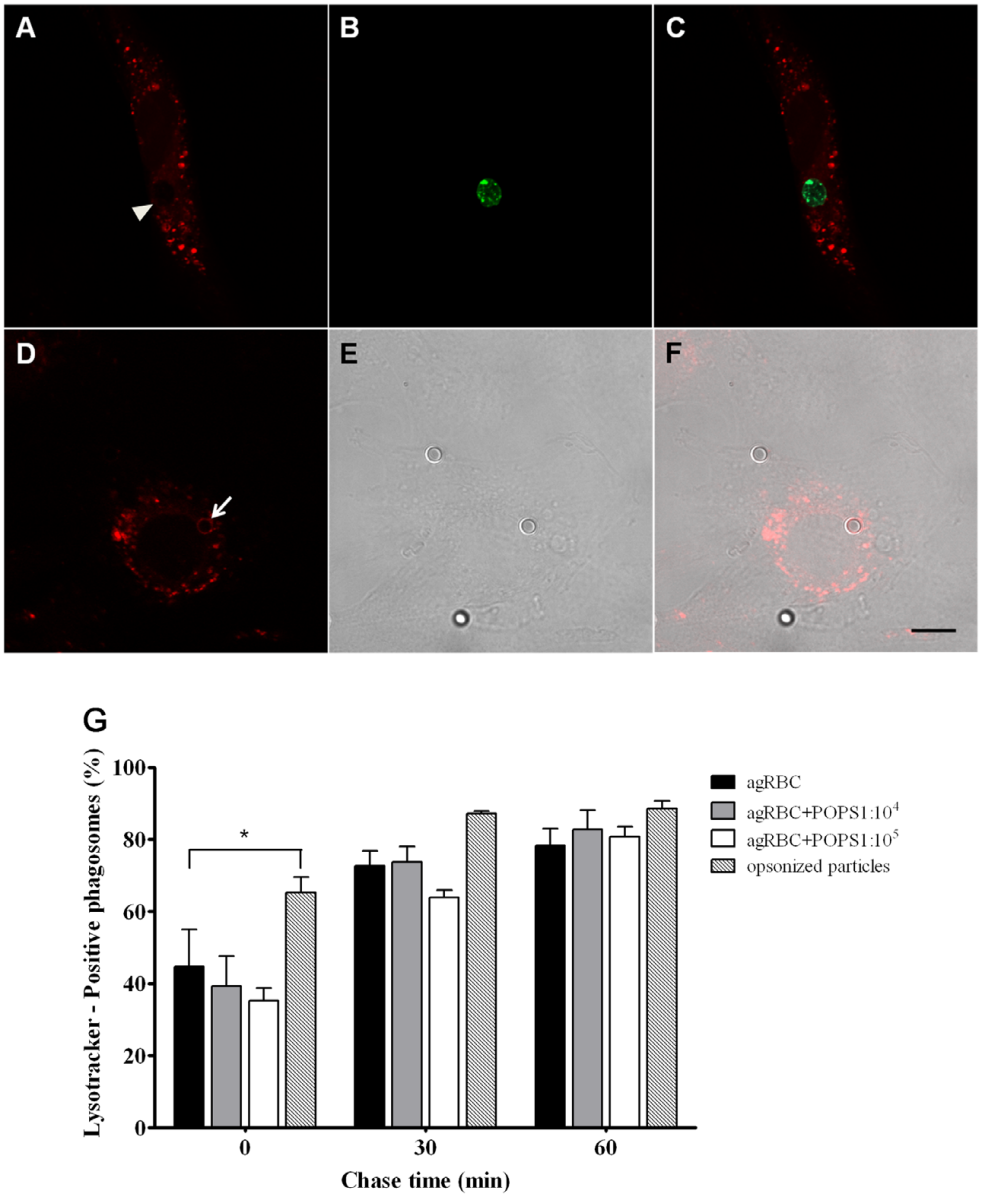
Acidification of phagosomes containing different phagocytic particles. Phagosome acidification was assessed with Lysotracker red. **A**) Lysotracker staining of a SMC containing a negative phagosome. **B**) Engulfed agRBC stained with CFSE. **C**) Corresponding merged image. **D**) Lysotracker staining of SMC stably expressing FcRγ-IIA with a Lysotracker-positive phagosome. **E**) Corresponding DIC image. **F**) Corresponding merged image. Arrow indicates Lysotracker-positive phagosome and arrowhead indicates a negative phagosome at 0 min chase time (30 min pulse). Bars, 10 µm. **G**) Quantification of the Lysotracker-positive phagosomes. Wild type and engineered SMCs were exposed to different phagocytic particles for 30 min and then chased for the times indicated in the graph abscissa. Before image acquisition, the cells were incubated with Lysotracker for 5 min. Data shows the percentage of Lysotracker-positive phagosomes and are means ± SEM of, at least, three independent experiments. At each time point 100 phagosomes were analyzed. *, p < 0.05 comparing differences between Lysotracker-acquisition by phagosomes with agRBC and with IgG-opsonized latex beads.

Wild-type SMC or SMC stably expressing the Fcγ-RIIA were plated in 24-well plates at 30 × 10^3^ cells/well and grown on glass coverslips for 24 h. The culture medium was replaced with CO_2_-independent medium (RPMI-1640 Modified medium, Sigma), and then the different phagocytic particles were added. The onset of phagocytosis was synchronized by centrifugation and then cells were incubated at 37°C for different time points.

For binding experiments, phagocytic particles were added to SMC cells, centrifuged and kept on ice at 4°C for different time periods. Then the cells were shifted to 37°C for 35 s before 5 vigorous washes with ice-cold PBS. Cells were then fixed with 4% paraformaldehyde (PFA).

**Figure 7:**
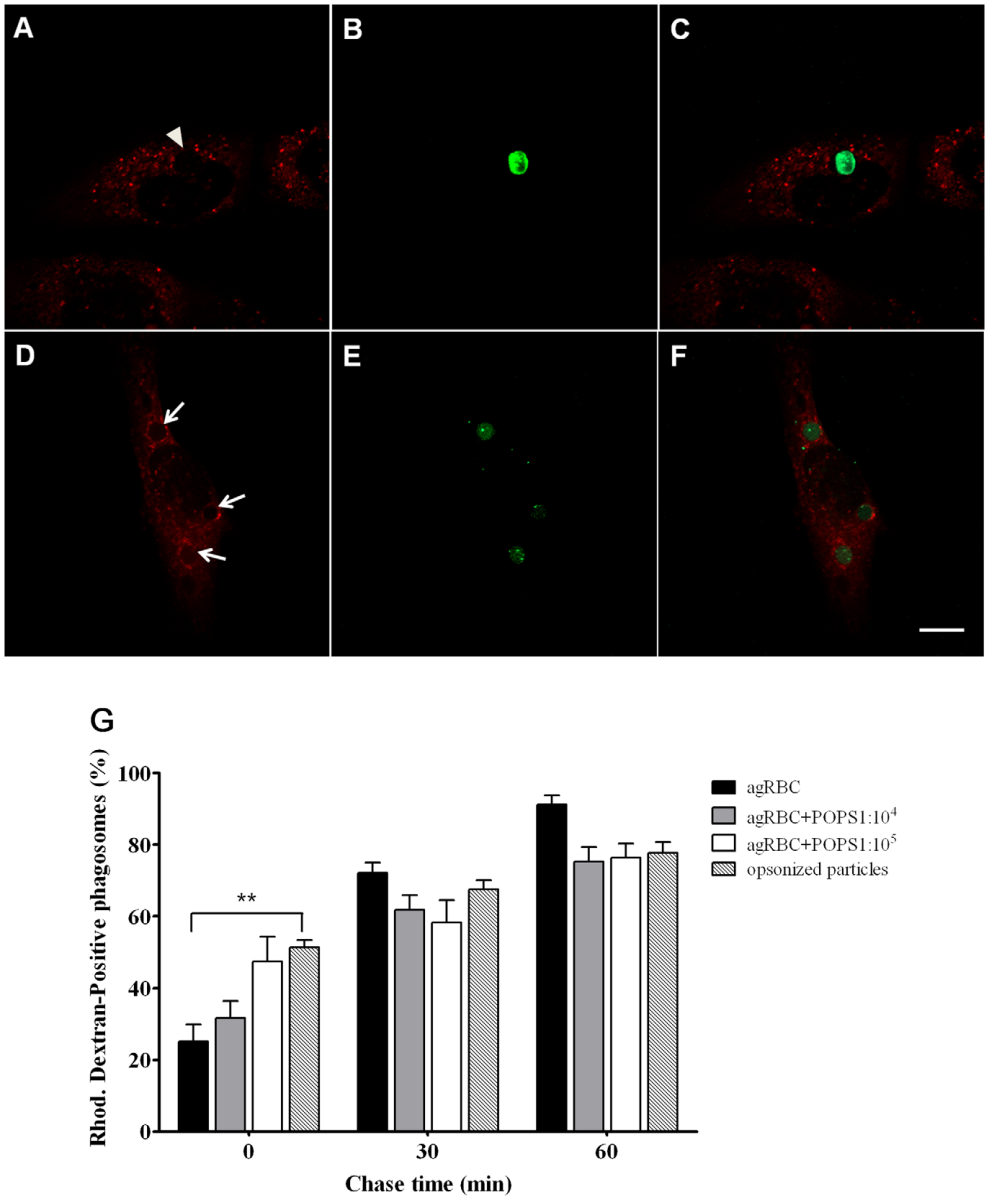
Interaction of phagosomes containing different phagocytic particles with lysosomes. Before challenging SMC with phagocytic particles, these cells were incubated with Rhodamine-Dextran and then chased to label lysosomes as described in Experimental Procedures. Phagolysosome formation was then analyzed by looking at the phagosomal fluorescence acquisition. **A**) Representative image of lysosomal staining with Rhodamine-Dextran in a SMC containing a Rhodamine-Dextran-negative phagosome. **B**) Engulfed agRBC stained with CFSE. **C**) Corresponding merged image. **D**) Representative image of lysosomal staining with Rhodamine-Dextran in a SMC stably expressing the FcRγ-IIA containing three Rhodamine-Dextran-positive phagosomes. **E**) Engulfed shRBCs stained with CFSE. **F**) Corresponding merged image. Arrows indicate three positive phagosomes and arrowhead indicates a negative phagosome at 0 min chase time (30 min pulse). Bars, 10 µm. **G**) Quantification of Dextran-positive phagosomes. Wild type and engineered SMCs were exposed to different phagocytic particles for 30 min and then chased for the times indicated in the graph abscissa. Data shows the percentage of Rhodamine-Dextran-positive phagosomes and are means ± SEM of at least three independent experiments. At each time point 100 phagosomes were analyzed. **, p < 0.01 comparing differences between Rhodamine-Dextran-acquisition by phagosomes with agRBC and with IgG-opsonized RBC.

For the Phagocytic Index (PI) the cells were challenged for different time points with the different phagocytic particles and after lysing the non-internalized RBCs, the cells were fixed with 4% PFA for 30 min, permeabilized with 0.1% Triton X-100 and then stained for actin with Rhodamine-Phalloidin (1∶500 dilution) for 30 min at RT, while nuclei were stained with DAPI (30 nM) for 20 min at RT.

Pulse-chase experiments were performed to assess phagosome maturation. Briefly, after the pulse (during which phagocytic particles were allowed to be in contact with phagocytes), the non-internalized RBCs were lysed or the beads stained (on ice) with an anti-human antibody conjugated with a fluorophore. Then the cells were shifted to 37°C for different time points (chase).

### Recycling of YFP-GL-GPI from the Phagosomal Membranes Back to the Plasma Membrane

For the recycling of the plasma membrane marker glycosyl-phosphatidyl-inositol-anchored yellow fluorescent protein (YFP-GL-GPI), wild-type SMC and the variant SMC stably expressing Fcγ-RIIA were infected with adenoviruses expressing the YFP-GL-GPI for 1 h at 37°C. After overnight incubation the cells were used for the phagocytosis experiments.

### Fluorescence and Confocal Microscopy

To estimate phagosomal pH, cells were allowed to internalize particles and then 50 nM LysoTracker Red was added. Labeling was terminated after 5 min by placing the cells on ice. After incubation, cells were washed, fixed in 4% PFA and analyzed by fluorescence microscopy to determine the percentage of LysoTracker-positive phagosomes.

To stain the lysosomes cells were plated and, after adhesion, incubated with Dextran Tetramethylrhodamine conjugate (1 mg/mL) in a serum-free media for 24 h. Then the cells were washed and chased in normal culture medium for 2 h at 37°C to ensure lysosome delivery. After lysosome staining, phagosomal maturation assays were performed as described above.

The protocols for immunostaining of Early Endosome Antigen-1 (EEA-1) [38] and lysobiphosphatidic acid (LBPA) [40] have been detailed in the respective references. The antibody dilutions used were EEA-1 (1∶50) and LBPA (1∶30). Fixed samples were analyzed by using the LSM 510 laser scanning confocal microscope (Zeiss) with a 63× oil immersion objective. Digital images were analyzed by using LSM Image Browser.

### Statistical Analysis

Student’s two-tailed t tests were performed to assess the significance of differences. Data shown represent means ± standard error of the *mean* (SEM) from at least three independent experiments.

## Results

### Generation of agRBC and their Enrichment in PS as Apoptotic Cell Models for Phagocytosis and Phagosomal Maturation Studies

RBCs are very good phagocytic models since they offer many advantages compared to other cell types: 1) RBCs cannot bind to phagocytes without previous modification on their surface; 2) they are easily induced to undergo an apoptotic-like process, termed eryptosis, mimicking senescent cells; 3) the plasma membrane levels of PS can be manipulated by incubating the cells with PS-liposomes; 4) RBCs can also be opsonized with antibodies and then used to study Fc-mediated phagocytosis; and 5) by applying a hypotonic shock the distinction between attached RBCs and those that have been internalized is easily done (see Fig. 1A).

We have, therefore, generated agRBC and used them as our apoptotic phagocytic model. Indeed, although RBCs cannot undergo apoptosis because they lack a nucleus, mature RBCs can undergo a rapid self-destruction process sharing several features with apoptosis, including cell shrinkage, plasma membrane microvesiculation and PS externalization, leading, in the presence of phagocytic cells, to their ingestion [41], [42]. To evaluate aging of RBCs we used the FITC-labeled Annexin-V, which recognizes and binds PS head group in the outer leaflet of the plasma membrane, and then performed flow cytometric analysis on these cells. When RBCs were incubated in PBS at 37°C for 4 days, PS translocated efficiently from the inner to the outer leaflet of the plasma membrane (Fig. 1B) and 88.9±3.5 % of cells were positive for Annexin-V. In contrast, less than 1% of freshly isolated RBCs (native RBC) were positive for Annexin-V and the fluorescence intensity was negligible (<3 %). The values obtained for ag- *versus* native-RBCs were within the range reported previously by others [5].

Data from many different laboratories have suggested that loss of phospholipid asymmetry and external expression of PS are required for recognition of apoptotic cells by macrophages and other phagocytes [43], [44]. However, the literature is not clear on whether more PS at the surface of apoptotic cells could control phagocytosis or phagosomal maturation. Therefore, we decided to load agRBC with more PS by incubating agRBC with PS liposomes at different cell:POPS (RBC/PS molecule) ratios. Although, there was a wide range of individual variation in the incorporation of PS, when agRBC were incubated with different ratios of RBC:POPS, for 24 h, the percentage of Annexin-V positive cells increased. By comparing with the agRBC we observed a ∼5 and ∼8 times enrichment in PS for the ratio of 1∶10^4^ and 1∶10^5^ (judged by the values of the mean fluorescence intensity), respectively (Fig. 1B) but no enrichment was obtained with the ratio 1∶10^3^. This last ratio was, therefore, ignored in further experiments.

To exclude that during all RBC treatments the integrity of their plasma membrane was not affected, the cells were incubated with Trypan blue (a vital dye that is negatively charged and does not interact with the cell unless the membrane is damaged). During aging or incubation of the agRBC with the three different cell:PS ratios no Trypan blue-positive RBC were observed (results not shown), suggesting that the integrity of the plasma membrane was not affected by any of the treatments.

Once the phagocytic models were characterized, we looked at the effect of PS loading in the outer leaflet of agRBC in binding and phagocytosis as a function of time in the smooth muscle cell line. SMCs, being of a non/myeloid origin, are classified as non-professional phagocytes. They are nevertheless capable of engulfing apoptotic cells (see below).

### Enrichment in PS Affected Neither Binding nor Phagocytosis of agRBC

Apoptosis of SMCs has been demonstrated to occur *in vitro* and *in vivo* and their uptake by adjacent normal SMC appears to be specific. This cell type has the ability to bind and ingest apoptotic bodies due to exposure of PS in the latter, and is an important participant clearance of cells fated to die in diseases such as atherosclerosis. In addition, besides macrophages, SMCs represent the major phagocytic population in the vessel wall [5]–[7].

PS has traditionally been referred to as the “eat-me” signal for receptor-mediated phagocytosis of apoptotic cells. However, the effect of PS loading into apoptotic particles in binding, phagocytosis and phagolysosome biogenesis was never addressed. Thus, here we have analyzed the relationship between PS loading of agRBC in binding and phagocytosis by SMCs.

To study binding of agRBC and agRBC loaded with PS these particles were labeled with the fluorescent dye CFSE and fixed with 0.2% glutaraldehyde. Then the different models of RBC (3×10^5^ cell/well) were incubated with SMCs for different time points, at 4°C, as shown in Fig. 2A. Unbound cells were removed by extensive washing with cold medium, and the cells were fixed with 4% PFA. After fixation and staining the cells for actin, the bound RBCs were counted under a confocal microscope. In contrast to native RBCs (result not shown), agRBCs and PS enriched agRBC associated with SMCs. Binding for all particles was time- and treatment- independent. The results obtained with agRBC and agRBC loaded with PS were very similar, suggesting that PS enrichment was not having any effect in the association of the phagocytic particle to the phagocyte. To validate our assay, we also incubated native RBCs enriched in PS with SMCs, as described above, at 4°C. As described by other groups [44] these RBCs were efficiently bound to SMCs. Our results suggest that PS exposure is sufficient for binding, but enrichment of the plasma membrane with more PS does not imply more binding.

Binding of an apoptotic particle to the phagocyte receptors may result in phagocytosis. To measure phagocytosis, human agRBC loaded, or not, with PS were labeled with CFSE, as described before, and incubated with SMC for different time points at 37°C as shown in Fig. 2B–G. After phagocytosis, the cells were fixed, permeabilized and stained with DAPI and Rhodamine-Phalloidin to visualize the cortical actin (illustrated in Fig. 2D–G). In these studies, the same number (3×10^5^ cell/well) of phagocytic particles was added per different experimental conditions. Phagocytosis phenotypes were quantified by averaging the percentage of SMC that have internalized phagocytic particles, which are reported as Phagocytic Index (PI). SMC engulfed all models of apoptotic cells tested (Fig. 2B–G). The PI ranged from 8.64±1.31 to 10.25±1.41 for agRCB and agRBC+POPS (1∶10^5^ ratio), respectively at 30 min of phagocytosis. Again, similar to what was described for the binding experiments, the rate of phagocytosis was independent of incubation time (at least for the times we tested) and PS concentration (Fig. 2B). The average number of particles engulfed by SMC was independent of the phagocytic particle, being between 1 and 2 (Fig. 2C). The native RBC enriched in PS, that were efficiently bound to SMC and as was described by others [44], were not engulfed (result not shown).

There were some quantitative differences between binding and the PI that were performed at different temperatures 4 and 37°C, respectively. To explain this discrepancy we can envision the following scenarios: i) appearance of more phagocytic receptors, via exocytosis, at the plasma membrane at 37°C; ii) further modification of agRBC, with the appearance of more “eat me signals” also at 37°C.

In conclusion, our results showed that around 5 to 8 times enrichment of agRBC in PS is neither affecting binding nor phagocytosis suggesting that probably the agRBC has enough PS to saturate all the PS-receptors at the plasma membrane of the SMCs.

An interesting feature of phagosomes is that they are organelles unable to perform their main task, the killing and degradation of microorganisms and apoptotic cells, immediately after formation at the plasma membrane. Indeed, the acquisition of phagosome functional properties depends on complex sets of interactions with various cellular organelles, leading to the biogenesis of phagolysosomes. Thus, we next assessed the role of PS loading in phagosomal maturation and compared it with the maturation of phagosomes containing IgG-opsonized inert particles.

### Phagosomes Containing IgG-opsonized and agRBC Particles Mature At Different Rates

The efficient particle digestion and processing requires dramatic remodeling of the phagosomal membrane, lumenal contents and the progressive acidification of its lumen, a process known as phagosomal maturation. This process culminates with fusion with lysosomes and subsequent phagolysosome formation [28], [45]. In order to check whether the amount of PS exposed on cells undergoing apoptosis could affect maturation, we decided to follow the maturation process of phagosomes containing the different phagocytic models generated above, by looking at the acquisition of endocytic markers, characteristic of different stages of maturation and acidification. The maturation kinetics of these phagosomes was compared with those phagosomes carrying IgG-opsonized particles, which are recognized via the well described Fcγ-RIIA. Because SMCs are non-professional phagocytes and do not express the Fcγ-RIIA in normal conditions, we used a retroviral system to generate an engineered SMC line stably expressing the Fcγ-RIIA, an abundant, widely expressed and highly studied phagocytic receptor. As phagocytic models we used shRBC and inert latex beads opsonized with antibodies. To synchronize phagocytosis the phagocytic particles were added to SMC and to the parental cell line (SMC stably expressing Fcγ-R) and submitted to a short spin. Then the cells were shifted to 37°C and cells were allowed to internalize particles for 30 min (pulse time). This time was chosen according to results obtained previously. After phagocytosis, the cells were shifted to 4°C and the non-internalized RBC lysed or the latex beads stained with secondary antibodies conjugated with a fluorophore. The cells were shifted again to 37°C to allow maturation of the new formed phagosomes. Thus, we performed pulse (30 min  =  0 min chase)/chase experiments to assess phagolysosome biogenesis. After each time-point investigated, depending on the marker, cells were fixed, immunostained (or not, as required) and analyzed by confocal fluorescence microscopy. A phagosome was considered positive for a given marker when a fluorescent ring was observed around the engulfed particle. In the case of latex beads that were internalized after the pulse (made visible by labeling with secondary antibodies as described above), they were excluded to ensure that we were just following the maturation of phagosomes formed during the first 30 min of phagocytosis. By independently measuring, as a function of time, the acquisition of endocytic markers, recycling and phagosomal acidification under identical experimental conditions in pulse-chase experiments we could decouple internalization from maturation.

To track the association of the nascent phagosomes with early components of the endocytic pathway, the recruitment of the Rab5-effector EEA-1, responsible for tethering early endosomes to nascent phagosomes, was assessed by immunofluorescence. For all phagocytic particles, EEA-1 association with phagosomal membranes was transient (Fig. 3). So the phagosomes acquired the marker at early chase times and lost it in the course of time. As shown in Fig. 3G, shortly after particles ingestion (0 min chase), a majority of phagosomes containing IgG-opsonized particles, agRBC and agRBC enriched in PS (1∶10^5^ ratio) associated with EEA-1 (45±1.53%, 53.7±7.4% and 44±4.5%; respectively). For the same time point the percentage of EEA-1 positive phagosomes carrying agRBC enriched in PS (1∶10^4^ ratio) was lower when compared with the other phagosomes although not significantly so.

It is generally considered that phagolysosome biogenesis involves not only fusion with components of the endocytic pathway, but also fission events and recycling of plasma membrane components. Thus, we next examined the recycling of phagosomal components back to the plasma membrane, which is necessary for their constant remodeling and progressing of the maturation process. In order to follow trafficking of plasma membrane components, phagocytes were infected with adenovirus expressing the marker YFP-GL-GPI, and its recycling was assessed by looking at the elimination of the marker from phagosomal membranes in time. As shown in Fig. 4 the plasma membrane protein YFP-GL-GPI was eliminated/recycled, with time, from all phagosomes. However, as shown in the graph (Fig. 4G) and illustrated in the Fig. 4A–F, the recycling of YFP-GL-GPI from phagosomal membranes back to the plasma membrane was faster in phagosomes containing IgG-opsonized particles compared with the other phagocytic particles. This effect was more pronounced at 15 min chase where the percentage YFP-GPI-positive-phagosomes was significant higher for agRBC than for opsonized shRBC (61.0±5.5% and 37.0±6.2%; p<0.05, respectively), suggesting that the recycling is delayed in phagosomes containing senescent particles. Since recycling of components of phagosomal membranes is crucial for phagosomal progression we looked then at the acquisition of Multi Vesicular Bodies (MVB) /late endosomal markers to confirm that the kinetics of phagosomes containing senescent RBC and opsonized particles mature at different kinetics. The interaction of the different phagosomes with MVB/late endosomes was assessed by looking at the acquisition of LBPA. As shown in Fig. 5G and illustrated in Fig. 5A–F, phagosomes containing IgG-opsonized particles acquire LBPA much faster that the other phagosomes containing senescent particles with different levels of PS. The difference was more notorious at 0 and 45 min chase time. Once more, maturation kinetics of phagosomes sheltering agRBC was not very affected compared to those with PS-enriched agRBC.

Phagosomal maturation is accompanied by a gradual and profound decrease in pH levels, reported to be as low as 4.5 within the phagosome. Lysotracker probes, fluorescent dyes that have been extensively shown to be distributed in acidified compartments within a cell, e.g. lysosomes and late endosomes, were used to quantitatively assess phagosomal lumen acidification [44]. Thus, after performing the pulse-chase experiments we monitored phagosomal acidification by incubating living SMCs with Lysotracker for 5 min at 37°C. Phagosomal acidification was quantitatively assessed with fluorescent microscopy by determining the percentage of phagosomes that co-localized with Lysotracker red. Again, phagosomes containing IgG-opsonized particles acidified more rapidly than those carrying apoptotic models, an effect that was more evident at the beginning (0 min) of the chase period (Fig. 6). With the time the difference in acidification was attenuated.

The last event of phagosomal maturation includes fusion with lysosomes and consequent phagolysosome formation. In absence of good anti-LAMP antibodies for immunofluorescence for rabbit cells, we decided to label the lysosomes with Dextran conjugated with Rhodamine. For this purpose wild-type and SMCs expressing the Fcγ-RIIA were preloaded with fluorescent Dextran in serum-free media for 24 h followed by 2 h chase to ensure that all internalized dextran had reached the lysosomes. Then the different phagocytic models were added to the phagocytes and normal pulse-chase experiments were performed. As shown in Fig. 7, phagolysosome formation was faster for opsonized cargo. This effect is more notorious and significant at 0 min chase time. At later time points, phagosomes containing opsonized particles reach the same level of maturity as those containing apoptotic cells. Interestingly, for all cases phagosomal acidification occurs prior to lysosomal fusion.

Except for EEA-1 acquisition, all the other markers tested showed, clearly, that different phagocytic particles, internalized by different receptors generated distinct phagosomes with different maturation kinetics. The results obtained for EEA-1 acquisition can be explained, in part, by the fact that the differences in phagosomal maturation kinetics were only delays and EEA-1 associates with phagosomal membranes only for few minutes. Thus, due to the short association of this endosomal marker with phagosomal membranes delays in phagosomal maturation are very difficult to observe.

Furthermore, the differences in phagosome maturation cannot be attributable to differences in cell type (Figure S1).

## Discussion

Little is known about how the final step of apoptotic cell clearance is regulated and more importantly how it differs from the processing of classically opsonized or microbial cells. Here, we tested the hypothesis that the particle itself can influence the intracellular trafficking of its phagosome inside a phagocyte. Especially, in this study, we characterized the maturation of phagosomes containing agRBC and the effect of PS enrichment of the agRBC in phagosomal maturation. The maturation of phagosomes containing agRBC was compared with that of IgG-opsonized particles containing phagosomes in a non-professional phagocyte cell line.

The phagocytic particles used in this work are recognized by different receptors. The apoptotic cells are likely to be internalized by many different receptors that are believed to function cooperatively [12]. The IgG-opsonized particles are internalized via a single receptor, the FcγR, one of the best and most studied phagocytic receptor, also known to be tightly coupled to the production and secretion of pro-inflammatory molecules such as reactive oxygen intermediates and arachidonic acid metabolites [46], [47]. More and more data support the notion that different phagocytic receptors send different signals to the actin cytoskeleton and initiate different mechanisms of internalization [48], [49]. Apoptotic signals stimulated membrane ruffling and formation of large and spacious fluid-filled vesicles; while Fc-receptor stimulation drove pseudopod extension from the phagocytic cell, resulting in a smaller tight fitting phagosome without any extracellular fluid. Indeed, such differences reinforce the idea that phagocytic particle properties can determine the complexity of the actin arrangement that must be created to dictate membrane remodeling over the target, phagosome formation and, perhaps, further phagosome interactions [1], [50], [51].

All events associated with clearance of apoptotic cells are extremely organized and elaborated [52]. Any disturbance of this refined process can be translated into different disease states linked to inflammation and autoimmunity [53]. Although, a lot of efforts have been dedicated to the role of receptors and ligands involved in recognition and post-engulfment consequences, the maturation of apoptotic cells containing phagosomes in mammalian cells has remained elusive [12]. Phagosomes containing apoptotic cells undergo maturation to generate phagolysosomes, in which cell corpses are degraded but the regulation of the maturation process is poorly understood. Several studies establish that the clearance of engulfed apoptotic bodies through phagosome maturation and fusion with lysosomes follows a path generally similar to that of other endosomes and phagosomes, but with some specific features. Recent studies in *C. elegans* have identified key factors involved in this maturation process, including Rab GTPases, PI3-kinases, and components of the HOPS complex. It is also noteworthy that components required for phagosome maturation, such as Rab5, also contribute to engulfment itself and this outcome was never observed in Fc-mediated phagocytosis [26], [27], [30], [32], [45], [54]–[57].

Here, the maturation of apoptotic cells containing-phagosomes was assessed by analyzing the time course of acquisition of early, late and lysosomal markers as well as by the loss of YFP-GL-GPI (a plasma membrane marker) and by acidification. To our knowledge this is the first time that such a systematic and comprehensive study has been done in mammalian cells. The rates of phagosome-lysosome fusion vary depending on the nature of the ingested particle. During Fc-mediated phagocytosis around 50% of the phagosomes have fused with lysosomes within 30 min (0 min chase). In contrast, for the same time point only 25% of the phagosomes containing agRBC have fused with lysosomes. Excluding the interaction with early endosomes, this trend was observed for all markers assessed, including recycling of plasma membrane components and acidification. This outcome, *i.e.*, phagosomes containing apoptotic cells maturing more slowly than those carrying opsonized particles, is not surprising, since intuitively, we may imagine that the fight against potentially harmful invaders is something that the body needs to face more aggressively, which is reflected in agility for a prompt destruction. On the other hand, apoptotic cells do not represent an eminent danger, since their effective removal is something that occurs almost constitutively in the body, illustrated by the negligible number of apoptotic bodies normally seen in damaged tissues [58], [59]. These observations indicate that phagocytic targets can differentially affect the maturation rate, perhaps, through their phagocytic receptors or other host-cell factors.

However, Erwig and colleagues [25], in contrast with our results, showed that phagosomes containing apoptotic cells matured more rapidly than those containing opsonized cells in primary macrophages, macrophage cell lines and fibroblasts and this effect was independent of the phagocyte species or the ingested target cell. This discrepancy, in our opinion could have two different explanations: i) differences between the phagocytes or the models of phagocytic particles used; and ii) experimental conditions used to assess phagosomal maturation. We would like to emphasize that in our case the transport of phagosomes within the cells was followed by pulse-chase experiments. This experimental approach allows the decoupling of particle internalization from the kinetics of both phagosomal acidification and phagosomal-endosomal/lysosomal fusion. In contrast, Erwig and colleagues [25] followed the acquisition of late endocytic markers and acidification at different times of internalization and thus, did not discriminate between the internalization and maturation of the phagosomes containing apoptotic cells. In our opinion this difference, in the experimental protocol, can explain, at least in part, the apparent discrepancy between their results and ours. In addition, our data was justified by a rigorous and meticulous process of marking and analysis of different stages of maturation, starting with sorting/early endosomes and culminating with lysosome interaction.

Finally, we addressed how the amount of PS exposure on the surface of the cell corpse can affect its engulfment and maturation. For this purpose, we generated agRBC enriched in different amounts of PS. The role played by PS in phagocytosis of cells programmed to die is essentially linked to recognition and receptor engagement, in these circumstances, working as an “eat-me” signal. However, internalization by itself appears to involve and require additional ligands on apoptotic cells, PS alone being insufficient to mediate phagocytic uptake [44]. However, we did not observe any significant effect in engulfment of agRBC enriched in PS or in posterior phagosome maturation suggesting that beyond a certain threshold the increase of negative charges at the surface of apoptotic cells by the incorporation of PS, a critical eat me signal, does not have any effect on the parameters measured in this work. While the literature suggests that the rate at which phagosomes mature may be related to the nature of the interaction between the particle surface and the phagosomal membrane, our results show that there may be a saturation threshold of PS-receptors at the surface of the SMCs. The types and abundance of receptors capable of binding PS at the surface of SMC, to our knowledge, have still not been characterized. Another open question is whether the oxidized PS (a process that seems to occur during apoptosis) is also critical to induce apoptotic cell phagocytosis [60], [61].

Our data show that the interactions between the phagocytic models and the endocytic machinery of the SMCs are significantly different in their kinetic characteristics. These findings can be relevant since the understanding of how defects in apoptotic corpse removal translate into disease states is not completely understood and enhanced phagocytosis of apoptotic cells may be exploited for therapeutic gain [62]. Furthermore, studies using non-digestible latex beads have shown that the inability to degrade a target can result in decreased uptake [6]. The challenges ahead include the identification of critical players as well as the signalling pathways that orchestrate the different stages of engulfment and maturation. It is already known, by studies of apoptotic cell removal in mammalian macrophages, that RhoA and ERM (ezrin–radixin–moesin) proteins have a role in the timely recruitment of Rab7 to the phagosome while no effect was observed on maturation of phagosomes containing IgG-opsonized particles [25]. These differences might be closely related to the different immunological responses induced by distinct phagocytic targets. Studies focusing on the degradation of apoptotic cells may provide new platforms for investigating the mechanisms underlying the differential processing of different phagocytic targets.

## Supporting Information

Figure S1 **Phagosomal maturation kinetics of IgG-opsonized particles and agRBC in wild-type and Smooth Muscle Cells stably expressing the FcγR-IIA is similar.** The stably expression of the FcγR-IIA in wild-type SMC does not change YFP-GL-GPI recycling and acquisition of LBPA of phagosomes containing IgG-opsonized particles and agRBC. **A)** Quantification of the YFP-GL-GPI-positive phagosomes. **B)** Quantification of the LBPA-positive phagosomes. The results are means ± SEM of, at least, three independent experiments. Samples were analyzed by fluorescence confocal microscopy. At each time point 100 phagosomes were analyzed. *, p < 0.05; **, p<0.01; ***; p<0.001 comparing differences between loss of YFP-GL-GPI or LBPA acquisition by phagosomes with agRBC and with IgG-opsonized RBCs. The experimental details have been described in the legends of the Figures 4 and 5.
(TIF)
