# Supplementary figures and images for: Comparison of the Kinetics of Maturation of Phagosomes Containing Apoptotic Cells and IgG-Opsonized Particles
Source: PLoS One. 2012 Oct 31;7(10):e48391. doi: 10.1371/journal.pone.0048391 (PMC3485219; doi:10.1371/journal.pone.0048391)

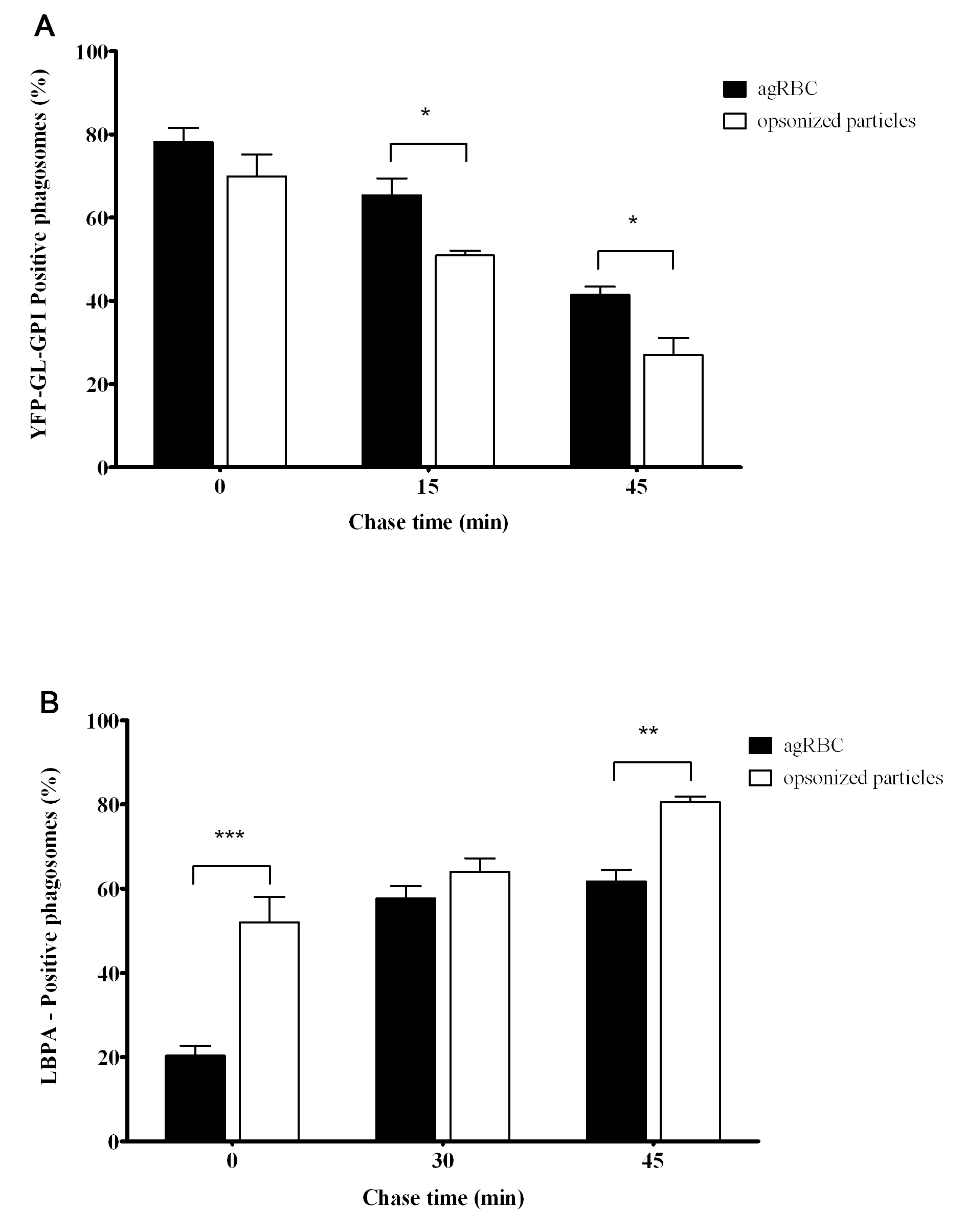

Supplement: Figure S1 — Phagosomal maturation kinetics of IgG-opsonized particles and agRBC in wild-type and Smooth Muscle Cells stably expressing the FcγR-IIA is similar. The stably expression of the FcγR-IIA in wild-type SMC does not change YFP-GL-GPI recycling and acquisition of LBPA of phagosomes containing IgG-opsonized particles and agRBC. A) Quantification of the YFP-GL-GPI-positive phagosomes. B) Quantification of the LBPA-positive phagosomes. The results are means ± SEM of, at least, three independent experiments. Samples were analyzed by fluorescence confocal microscopy. At each time point 100 phagosomes were analyzed. *, p < 0.05; **, p<0.01; ***; p<0.001 comparing differences between loss of YFP-GL-GPI or LBPA acquisition by phagosomes with agRBC and with IgG-opsonized RBCs. The experimental details have been described in the legends of the Figures 4 and 5. (TIF) [file pone.0048391.s001.tif]
